# Mediterranean Diet, Ketogenic Diet or MIND Diet for Aging Populations with Cognitive Decline: A Systematic Review

**DOI:** 10.3390/life13010173

**Published:** 2023-01-06

**Authors:** Paschalis Devranis, Εmilia Vassilopoulou, Vasileios Tsironis, Panagiotis Marios Sotiriadis, Michail Chourdakis, Michalis Aivaliotis, Magdalini Tsolaki

**Affiliations:** 1 1st Department of Neurology, School of Medicine, Faculty of Health Sciences, Aristotle University of Thessaloniki, AHEPA University Hospital, 54636 Thessaloniki, Greece; 2 Department of Nutritional Sciences and Dietetics, International Hellenic University, 57400 Thessaloniki, Greece; 3 Laboratory of Hygiene, Social & Preventive Medicine and Medical Statistics, School of Medicine, Faculty of Health Sciences, Aristotle University of Thessaloniki, 54124 Thessaloniki, Greece; 4 Basic and Translational Research Unit, Special Unit for Biomedical Research and Education, School of Medicine, Aristotle University of Thessaloniki, 54124 Thessaloniki, Greece; 5 Greek Alzheimer Association and Related Disorders, 54643 Thessaloniki, Greece

**Keywords:** Mediterranean diet, ketogenic diet, MIND diet, mild cognitive impairment, Alzheimer’s disease, dementia, aging, systematic review

## Abstract

(1) Background: Compelling evidence shows that dietary patterns can slow the rate of cognitive decline, suggesting diet is a promising preventive measure against dementia. (2) Objective: This systematic review summarizes the evidence of three dietary patterns, the Mediterranean diet, the ketogenic diet and the MIND diet, for the prevention of cognitive decline. (3) Methods: A systematic search was conducted in major electronic databases (PubMed, ScienceDirect and Web of Science) up until 31 January 2022, using the key search terms “Mediterranean diet”, “ketogenic diet”, “MIND diet”, “dementia”, “cognition” and “aging”. A statistical analysis was performed using RoB 2 and the Jadad scale to assess the risk of bias and methodological quality in randomized controlled trials. (4) Results: Only RCTs were included in this study; there were eleven studies (*n* = 2609 participants) of the Mediterranean diet, seven studies (*n* = 313) of the ketogenic diet and one study (*n* = 37) of the MIND diet. The participants’ cognitive statuses were normal in seven studies, ten studies included patients with mild cognitive impairments and two studies included Alzheimer’s disease patients. (5) Conclusion: All three dietary interventions have been shown to slow the rate of cognitive decline in the included studies. The Mediterranean diet was shown to be beneficial for global cognition after 10 weeks of adherence, the ketogenic diet had a beneficial effect for patients with diabetes mellitus and improved verbal recognition, while the MIND diet showed benefits in obese patients, improving working memory, verbal recognition, memory and attention.

## 1. Introduction

Dementia is the seventh leading cause of death worldwide, affecting approximately 55 million people; in the next decade, the number of dementia patients worldwide is estimated to increase by 50% [1]. The most common cause of dementia is Alzheimer’s disease (AD). The strongest genetic risk factor for AD is carrying the ε4 allele of the Apolipoprotein E (APOE) [2,3]. About 25% of the general population has at least one ε4 allele, with a three-fold increased risk of AD for heterozygotes and a nearly 15-fold increased risk for homozygotes [2]. Current pharmacological treatments for AD and dementia have proven to be ineffective [4]. Hence research efforts have shifted towards non-pharmacological treatments, especially in the earlier stages of dementia, and towards prevention strategies [5,6].

Mild cognitive impairment (MCI) is an intermediate state between normal cognition in healthy individuals and dementia and is characterized by a notable decline in cognitive abilities with the essential functional abilities being preserved [7]. Non-pharmacological therapies for MCI have been shown to have a preventive role in dementia and to stabilize cognitive decline [8,9,10,11]. These approaches focus on aggravating factors, such as cardiovascular disease (CVD), depression, hearing loss and brain injury [5]. There is an increased research interest in dietary interventions in dementia prevention. Among the various dietary patterns, the Mediterranean diet (MeDi) has been reported to reduce the risk of MCI and its progression to dementia [12,13].

MeDi is a dietary pattern that is common in countries bordering the Mediterranean Sea and is characterized by a high intake of plant-based food products, including fruits, vegetables, legumes, nuts and seeds, and whole grains. Olive oil is the principal fat used and is also added liberally to salads and meals. Fish and red wine are consumed in moderate amounts, while red meat, confectionery and highly processed foods are consumed infrequently [14]. Adherence to a MeDi has been associated with a reduced rate of cognitive decline in AD [15], improved overall cognition and episodic memory, and a lower risk of cognitive impairment and neurodegenerative diseases [16]. Oxidative stress and inflammation are known to exert significant deleterious effects on cognitive decline and brain ageing [17]. The bioactive dietary constituents of the MeDi, including phenolic compounds, have been shown to reduce neuroinflammation due to antioxidant actions [18]. Furthermore, MeDi adherence has been correlated with a reduced risk of coronary heart disease, hypertension, diabetes mellitus (DM), dyslipidemia and metabolic syndrome, which are all also associated with MCI, dementia and AD risks [19].

Although there is no strict definition of the MeDi [20], there are multiple scoring systems developed to measure MeDi adherence. The initial MeDi score of eight food items developed by Trichopoulou and colleagues [21] was revised to include fish as a ninth food item [22]. In this nine-point MeDi score, one point is given for above average consumption of each food item, ranging from the minimum, 0, to the maximum, 9. This approach has been further investigated by Martínez-González and colleagues using a Mediterranean Diet Adherence Screener (MEDAS), which initially included nine food items [23] and was then further revised to include fourteen food items [24]. This 14-point MeDi screening score ranges from the minimum, 0, to the maximum, 14, and one point for each consumed food item is given. A more detailed 55-point MeDi score has been developed by Panagiotakos and colleagues, taking 11 food items into account and distributing five points for each food item adhering to the MeDi, ranging from the minimum, 0, to the maximum, 55 [25]. Another approach is the Mediterranean Adequacy Index (MAI) which considers 18 food groups and their energy percentages of the total calorie intake [26]. To calculate the MAI, the sum of the energy percentages of ten typical MeDi food groups are divided by the sum of the energy percentages of eight non-typical MeDi food groups. All these scoring tools have been proven indispensable in evaluating one’s adherence to the MeDi and associated health benefits.

Previous systematic reviews and meta-analyses provide consistent evidence that MeDi adherence is associated with improved outcomes in cognitive function and performance as well as a reduction in cognitive decline compared to patients who do not follow a MeDi-type pattern [27,28,29].

The ketogenic diet (KD) is another dietary approach that has been correlated with reduced cognition decline in dementia patients and targets glucose metabolism, which is also known to be impaired in dementia patients [30,31,32]. AD patients have lower levels of brain insulin signaling and fewer brain insulin receptors, culminating in brain insulin resistance (IR) [33,34]. The KD, which is a high-fat, low-carbohydrate diet, is being used successfully in the management of drug-resistant epilepsy [35]. Different types of KDs exist, four of which include the classical KD, a KD with the use of medium-chain triglycerides (MCT), the modified Atkins diet and the low-glycemic-index diet. Depending on the type, a KD can induce various levels of nutritional ketosis, which restricts carbohydrate production and accelerates ketone production [36]. Typically, the blood concentration of ketones is below 0.3 mmol/L, higher levels of ketones are considered nutritional ketosis and the maximum blood concentration of ketones reached in a KD is 7–8 mmol/L [37]. The brain uses ketones as an alternative source of energy when glucose is not available as they can be converted to acetyl Coenzyme A (CoA). Entering the citric acid cycle, acetyl CoA undergoes oxidative phosphorylation and generates adenosine triphosphate (ATP) [38]. IR and DM are significant risk factors for AD [39], and therefore low-carbohydrate diets can be also beneficial in AD management [40]. In addition, MCTs have been suggested to improve cognition in patients with AD [41], and in particular, the non-carriers of the APOE ε4 allele [42]. MCTs are six to twelve carbons in length and are metabolized to acetyl CoA, resulting in ketogenesis and increased levels of beta-hydroxybutyrate (βHB). Because of the difficulties in maintaining a diet strict enough to achieve ketogenesis, MCT supplementation has been suggested, which has shown positive cognitive effects [43]. MCT intake, however, would entail an increased intake of saturated fatty acids, with an associated risk for inflammatory processes and cardiovascular health [44].

Another promising diet for dementia prevention is the Mediterranean-DASH (Dietary Approaches to Stop Hypertension) Intervention for Neurodegenerative Delay (MIND) diet, which is a hybrid of the MeDi and the DASH diet [45,46]. Whereas the DASH diet was previously developed to target hypertension and maintain cardiovascular health, it contains food items, such as butter, margarine and other dairy products, which are limited in the MeDi [47]. The MIND diet integrates food items with neuroprotective properties, such as olive oil, blueberries and oily fish. Furthermore, the MIND diet consists of 15 food components that are recommended in high or low amounts; in brief, it recommends plenty of vegetables, berries, nuts, olive oil, whole grains, fish and beans, a moderate amount of red wine, and restricted amounts of dairy products, red meat, fried food and sweets. According to the MIND diet score, a score of 0, 0.5 or 1 is given for each of the 15 food items of interest with a possible range from the minimum, 0, to the maximum, 15 [45,46].

The primary focus of the MIND diet is the prevention of dementia through the modulation of cardiovascular factors and increased neuroprotection exerted by the antioxidant and anti-inflammatory ingredients [45,46]. Studies on the MIND diet in dementia prevention are in agreement that a high adherence to the MIND diet is associated with better cognitive health and decreased dementia incidence [48].

Systematic reviews and meta-analyses have individually associated the MeDi, DASH and MIND diets with reduced cognitive decline and a lower risk of AD [12]. To our knowledge this is the first systematic review on the effects of the MeDi, KD and MIND diets on cognition.

The aim of this systematic review was to identify the components of each of the three dietary patterns, the MeDi, KD and MIND diets, that have been reported to be beneficial in improving cognition or slowing the rate of cognitive decline in the elderly.

## 2. Materials and Methods

### 2.1. Literature Selection Criteria

A systematic literature search was conducted in the electronic databases of PubMed, ScienceDirect and Web of Science. Publications including search terms of dietary interventions and cognitive status were selected for review, specifically MeDi, KD, MIND diets, cognition, aging, dementia, AD and MCI. Detailed search string is described in the Supplementary Materials. Only peer-reviewed publications written in English were included in the review. No date restrictions were set. An initial search was conducted, with a second search keeping the review up to date via 31 January 2022. Additionally, available reviews were screened, and selected publications were added for further analysis covering databases that were not included. This study was performed according to the Preferred Reporting Items for Systematic Reviews and Meta-Analyses (PRISMA) guidelines [49]. A PRISMA guidelines checklist is included in the Supplementary Materials. The present review does not have a prior protocol and has not been registered. All data is available by request.

### 2.2. Eligibility Criteria

Primarily, randomized controlled trials (RCT) with patients over 40 years old were included in this systematic review. For inclusion, all studies needed to report cognitive outcomes measured by at least one cognitive assessment tool after a period following each diet. All three criteria: dietary intervention, measurement of diet and measurement of cognitive status, had to be met in all the selected publications.

### 2.3. Study Selection

The online service of Rayyan was used for blinding the reviewers and the selection of the studies to be included [50]. Duplicate records were excluded. Titles and abstracts were screened for study eligibility, and full-text articles were reviewed by two reviewers for each diet. Cases of disagreement were discussed until consensus was reached. A third reviewer examined the studies in case of further disagreement.

#### 2.3.1. Outcomes

The primary outcome of the reviewed studies was the assessment of cognitive status in relation to dietary intervention. Cognitive status refers to patient being cognitively normal, or having MCI or dementia, as in AD. Assessment of cognitive status includes a variety of cognitive test batteries, most commonly the Mini Mental Status Examination (MMSE) [51] and the Montreal Cognitive Assessment (MoCA) [52].

#### 2.3.2. Data Extraction

When available, the following data were extracted from each study: (1) study design; (2) number of participants; (3) gender of participants; (4) age of participants; (5) cognitive status of participants at baseline; (6) cognitive test batteries; (7) duration of intervention; (8) outcomes of the study; (9) statistical significance of outcomes; (10) details of dietary intervention; (11) country of study; (12) health status of participants and other diseases; and (13) diet adherence.

#### 2.3.3. Quality Assessment

The studies included were assessed for methodologic quality and risk of bias by two independent reviewers. Disagreements were discussed and resolved by a third, more experienced reviewer. The Cochrane Collaboration’s tool, the second version of the Revised Cochrane Risk of Bias instrument for randomized trials (RoB 2), was used for the risk of bias evaluation of RCTs [53,54], whereas the Oxford quality-scoring system was used for the quality evaluation [55]. The RoB 2 tool was used to estimate the risk of bias in randomized studies that were assessed in terms of the following domains: (1) randomization process; (2) deviation from intended interventions; (3) missing outcomes data; (4) measurement of the outcome; (5) selective outcome reporting; and (6) overall bias. Each domain was considered to have a “low risk” of bias, be of “some concern” or have a “high risk” of bias. According to RoB 2, a result of “high risk” for any individual domain leads to an overall high risk of bias, while a result of “some concern” in any individual domain is judged as an overall risk of “some concern”. If multiple domains were problematic, the confidence in the outcome decreased and the overall risk was considered as “high” [54]. The Oxford quality-scoring system consists of three items: randomization, blinding and description of patient withdrawals and dropouts, with scores ranging from zero to five points. A score of 0–2 indicates the RCT is of low quality, whereas a score of 3–5 indicates the RCT is of high quality [55]. The quality assessment of all the included studies is described in the Supplementary Materials.

#### 2.3.4. Data Synthesis

A meta-analysis was considered but it could not be performed due to the wide variation in the cognitive assessment tools used in the different studies that precluded the synthesis of the extracted data.

## 3. Results

The three types of dietary interventions, the MeDi, KD and MIND diets, were reviewed separately. No study was found to compare the outcome of the three diets in cognition.

**Figure 1:**
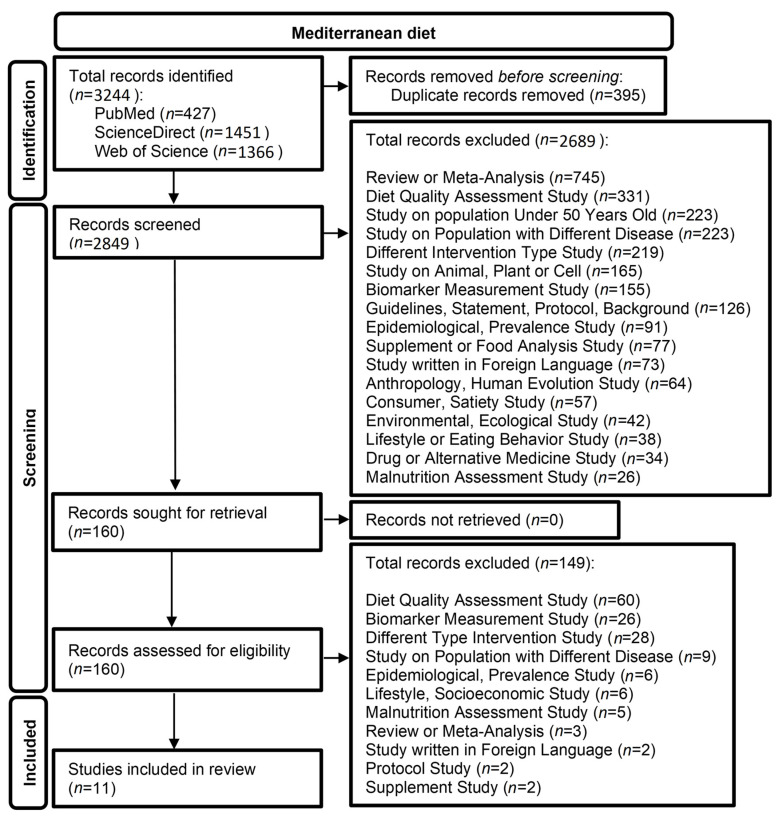
Flow chart of the literature search for articles on the Mediterranean Diet (MeDi).

In the search that was conducted, 3244 publications were identified (PubMed: 427, ScienceDirect: 1451, Web of Science: 1366). After removing 395 duplicates, 2849 studies were screened and assessed for eligibility, of which 2689 were excluded. Of the remaining 160 studies, only 11 fit the criteria of a RCT with cognitive testing. The flow chart of the selection process for the studies on the MeDi is presented in Figure 1.

Table 1 and Table 2 show the quality assessment of included studies. Table 3 shows the main characteristics of the 11 MeDi studies, all of which were published between 2000 and 2021. The period of the MeDi interventions varied between 4 weeks and 6.5 years. The total number of participants was 2609; in five studies the participants had normal cognitive function, in two studies the participants were diagnosed with MCI, one study had mixed participants with normal cognition and participants with MCI, and one study had participants diagnosed with MCI in Parkinson’s Disease (PD). All but one study reported beneficial effects of the MeDi intervention on cognitive function, reaching statistical significance for at least one cognitive test in a subgroup of participants. The shortest one with a 4-week intervention did not report significant benefits of the MeDi on cognition.

Firstly, in a four-week RCT conducted by Hoscheidt and colleagues, 87 middle-aged adults with a mean age of 56.3 ± 5.1 years were randomized into either a MeDi group (44 participants) or a Western diet group (43 participants) [56]. Participants received a cognitive and clinical evaluation, with 56 participants evaluated as cognitively normal with a modified Mini Mental Status Examination (3MS) score of 97.3 ± 2.4, and 31 participants with MCI with a 3MS score of 95.5 ± 4.4, which differed significantly (*p* = 0.007). Compliance was assessed using a daily food record with an average of less than one non-compliant meal per week observed for each diet. Cognitive testing examined participants’ delayed episodic memory via story recall and the Buschke Selective Reminding Test (BSRT), and executive function via the Dot Counting Test (DCT). After the intervention, no individual comparisons between the groups and their cognitive status achieved significance. Only a three-way interaction of diet x cognitive status x time achieved statistical significance on the composite score of the cognitive assessment tools (*p* = 0.049).

In the MedLey study, an RCT was conducted for 6 months by Knight and colleagues, including 137 cognitively normal men and women aged 72 ± 4.9 years, who were randomly assigned to either a MeDi group (70 participants) or were asked to continue their usual diet (67 participants) [57]. Compliance was measured by a daily food check list, a food frequency questionnaire (FFQ), a three-day food record and a range of biomarkers, such as erythrocyte fatty acid composition, plasma carotenoids and urinary metabolites, achieving a reported compliance of 92%. There was a statistically significant improvement within groups for the Benton Visual Retention Test (BVRT) (*p* = 0.01), the Rey Auditory Verbal Learning Test (RAVLT) (*p* = 0.03) and the composite for memory (*p* = 0.05) which consisted of the RAVLT, the Forward and Backward Digit Span test (F&B-DS) and the Letter–Number Sequencing (LNS) test. There were no significant differences between the groups’ mean performances in all the cognitive assessment tests and within the group mean for the Stroop Test, initial and excluded Letter Fluency Tests (LFTs), the Tower of London planning test (TOL), and the Symbol Search and Coding test (SS&C).

Marsegli and colleagues conducted an RCT for 12 months analyzing 1144 relatively healthy, older adults with a mean baseline age of 70.9 ± 3.4 years and a mean Mini Mental Status Examination (MMSE) score of 28.3 ± 1.6, from five European centers in France, Italy, the Netherlands, Poland and the UK [58]. The participants were randomly allocated into two groups; one group with 571 participants was given a leaflet with the national dietary guidelines, and the other group with 573 participants followed the NU-AGE diet, which is an individually tailored Mediterranean-like diet targeting older adults. Adherence to the diet was measured by a NU-AGE index based on food-based dietary guidelines. The baseline adherence was 51.5% ± 10% and at the end of the intervention it was 65.9% ± 11% in the intervention group, while in the control group it was 52.7% ± 10% (*p* < 0.001). After the intervention, the mean overall difference between the scores of the cognitive tests in both groups did not reach statistical significance. A further analysis revealed that those with moderate or high adherence to the NU-AGE diet showed a significant improvement in global cognition (*p* = 0.046) compared to those with low adherence, measured by a composite of the MMSE and the Consortium to Establish a Registry for Alzheimer’s Disease neuropsychological battery (CERAD-NB), consisting of the Category Fluency test (CFT), the Boston Naming Test (BNT), the Word List Memory test (WLM), the Constructional Praxis Tests (CPT), and tests for episodic memory (*p* = 0.025), measured by a composite of the WLM and the Babcock Story Recall Test (BSRT). No difference was found in the Trail Making Test-part A and B (TMT-A&B), Digit Cancellation (DC) test and Pattern Comparisons Test (PCT).

The PREvención con DIeta MEDiterránea (PREDIMED)-NAVARRA trial is a nutritional RCT conducted in the Navarra province of Spain with a mean follow-up of 6.5 years. Three of its publications by Martínez-Lapiscina and colleagues were included in this review [59,60,61]. Participants had DM or ≥ three risk factors for CVD and were randomly allocated to three different groups. One intervention group followed a MeDi supplemented with 1L of extra virgin olive oil (MeDi-EVOO) per week; the second intervention group followed a MeDi supplemented with 30 g of raw, unprocessed mixed nuts (MeDi-MN) per day, specifically, 15 g walnuts, 7.5 g almonds and 7.5 g hazelnuts; and the third group, the control group, followed a low-fat (LF) diet. Adherence was measured by a 14-point MEDAS.

Initially, the cognitive effects and cognitive status of 522 community-dwelling participants with a mean age of 67.4 ± 5.7 years at baseline were investigated, with 224 participants in the MeDi-EVOO group, 166 in the MeDi-MN group and 132 in the LF group [59]. Unfortunately, the baseline cognitive performance or cognitive status of participants was not stated, as the normal cognitive performance or normal cognitive status is not given in a community-dwelling population. The five-year MEDAS was 10.5 ± 3.3 in MeDi-EVOO, 9.1 ± 4.9 in MeDi-MN and 5.8 ± 4.7 in LF, while the six-year MEDAS was 10.8 ± 2.7, 10.1 ± 4 and 6.3 ± 4.7, respectively (*p* < 0.001). The cognitive assessment included the MMSE and the Clock-Drawing Test (CDT). Participants in the MeDi-EVOO group had a significantly better performance in the MMSE (*p* = 0.04) and in the CDT (*p* = 0.02) in comparison to that of the LF group. After the multivariable adjustment, the participants of the MeDi-MN group had significantly higher mean scores in the MMSE (*p* = 0.02) and the CDT (*p* = 0.048) in comparison to those of the LF group. After the dietary intervention, the MeDi with EVOO group showed higher mean global cognitive function scores than those of the LF group on MMSE (*p* = 0.005) and on CDT (*p* = 0.001). The adjusted mean scores were also higher in the MeDi with nuts group than in the LF group on MMSE (*p* = 0.015) and on CDT (*p* = 0.048). It is reported that the presence of the ε4 allele decreased the MMSE score by 0.8 and the CDT score by 0.53, the female sex decreased these scores by 0.56 and 0.49 points, respectively, age decreased them by 0.09 and by 0.07 for each year of increase, respectively, and more education increased these scores by 0.16 and by 0.12, respectively. Additionally, it is reported that 60 participants developed MCI via the Peterson criteria and 35 participants were diagnosed with dementia via the Diagnostic and Statistical Manual of Mental Disorders, Fifth Edition, (DSM-IV) criteria after follow-up, without any further analysis.

An investigation of the diet–gene interactions was conducted for the CR1, CLU, PICALM and APOE genes [60]. The MeDi-EVOO and MeDi-MN groups were merged into one MeDi group with 381 participants. There was no statistically significant difference in cognitive performance when considering genetic variants. A further analysis revealed that carriers of a T minor allele for the CLU gene in the MeDi group performed significantly better in the MMSE on the likelihood ratio test (*p* = 0.04) with an increase of 0.97 points (*p* < 0.001) and in the CDT by 0.6 points (*p* = 0.001). A beneficial effect of the MeDi was observed also for the CR1 gene with an increase of 0.76 points in MMSE (*p* = 0.001) and 0.46 in CDT for participants without the A risk allele (*p* = 0.006). A significantly beneficial interaction between the MeDi and the PICALM gene was found for MMSE in participants without a T minor allele with an increase of 0.76 points (*p* = 0.02) and in participants with at least one T allele with an increase of 0.51 points (*p* = 0.046) and an increase of 0.59 points (*p* = 0.005) in the CDT. As for the APOE gene, the ε4 allele significantly decreased the scores by 0.96 points in the MMSE (*p* < 0.001) and by 0.5 points in the CDT (*p* = 0.007). Participants without a ε4 allele in the MeDi group had a significantly better performance in the MMSE by 0.56 points (*p* = 0.007) and in the CDT by 0.55 points (*p* < 0.001), while carriers of a ε4 allele had an even greater increase only in MMSE by 1.6 points (*p* = 0.04).

A further analysis of the effects on cognition and cognitive status was performed on a subgroup of 268 participants with a mean age of 67.3 ± 5.7 years: 91 in the MeDi-EVOO group, 88 in the MeDi-MN group and 89 in the LF group [61]. The cognitive assessment tools included the MMSE, the CDT, the Verbal Paired Associates Test (VPAT), the RAVLT, the Rey–Osterrieth Complex Figure (ROCF) test, the BNT, an animals fluency task (AFT), the F-A-S test, the F&B-DS test, the TMT-A&B, a similarities test and the Clinical Dementia Rating (CDR). The cognitive outcomes in all the cognitive tests did not differ statistically significantly between the groups. In the multivariable-adjusted model, participants of the MeDi-EVOO group significantly improved their scores in the MMSE, the ROCF, the F-A-S test and the F-DS (*p* < 0.05) test. The MeDi-MN group did not differ from control. As for the comparison of their cognitive status, at the 6.5-year follow-up, 34 participants were identified with MCI and 5 with dementia. The adjusted analysis revealed that the MeDi-EVOO group had significantly less incidences of MCI compared to the LF group (*p* = 0.02) with an odds ratio for MCI of 0.34 (*p* = 0.04).

In another PREDIMED RCT, conducted by Valls-Pedret and colleagues in the Barcelona-North center, the objective was to investigate the effect of these diets on cognitive function [62]. The study analyzed 334 cognitive healthy community-dwelling participants with a baseline mean age of 66.8 ± 5.6 years and a baseline mean MMSE score of 28.3 ± 1.4, who were randomly assigned to a MeDi-EVOO group (127 participants), to a MeDi-MN group (112 participants), or to a LF diet group (95 participants). Participants in the LF group were significantly younger (*p* = 0.005), had lower high-density lipoprotein (HDL) values (*p* = 0.01), had more dropouts (*p* = 0.004), more APOE ε4 alleles (*p* = 0.004) and worse MMSE scores (*p* = 0.006). The adherence according to the 14-point MEDAS was 8.5 ± 1.8 at the baseline and changed to a mean of 10.5 in the MeDi-EVOO group, 10.7 in the MeDi-MN group and 9.1 in the LF group at the endpoint, which showed a significant difference between the groups (*p* < 0.001). After a median follow-up time of 4.1 years, the memory composite comprising the RAVLT and VPAT scores was significantly improved in the MeDi-MN group in the fully adjusted model (*p* = 0.04). Moreover, the MeDi-EVOO group significantly improved in the fully adjusted model in the frontal composite comprising the F&B-DS and the Color Trail Test (CTT) scores (*p* = 0.004), as well as in the global cognitive composite comprising the scores of all neuropsychological tests, including the MMSE and AFT scores (*p* = 0.008). Participants in the LF group had significantly worse composites scores (*p* < 0.05). As for cognitive status, 37 incidences of MCI via the Peterson criteria and no incidence of dementia via the DSM-IV criteria were identified at the follow-up without significant differences between the groups (*p* > 0.05).

An Italian RCT by Mazza and colleagues investigated, in the context of a MeDi, whether a replacement of all vegetable oils with EVOO would improve cognitive performance in the elderly [63]. The study analyzed 110 individuals with a baseline mean age of 70 ± 4 years and a mean MMSE score of 24.6 ± 1.4. Although not stated in the publications, this MMSE score is in the range of MCI. Participants were randomly assigned to one of two dietary intervention groups for 12 months, either a MeDi group in which all vegetable oils were replaced by EVOO at a dose of 20–30 g per day (55 participants), or a control MeDi group with no such substitution (55 participants). Adherence to the MeDi was monitored by a 24 h recall and a 7-day food record to calculate the MAI which was at baseline 2.9 ± 1 and at the follow-up 3.2 ± 1 with a significant change in the EVOO consumption (*p* = 0.04). After 12 months, there was a significant improvement in the MMSE and in the Alzheimer’s Disease Assessment Scale-Cognitive Subscale (ADAS-Cog) in both groups (*p* < 0.001). Notably, there was a significant difference between the groups in the ADAS-Cog with a greater improvement in the MeDi-EVOO group in the adjusted model (*p* = 0.02). No differences were found in verbal fluency (VF).

In an RCT in Iran, Paknahad and colleagues investigated the effects of the MeDi on cognitive function in patients with MCI in PD (PD-MCI) [64]. A 10-week intervention trial analyzed 70 patients with a mean age of 59 ± 8.8 years and a mean Montreal Cognitive Assessment (MoCA) score of 19.4 ± 5.8; they were randomly divided into two similar groups, the MeDi group with 35 participants, and the control diet group with 35 participants. The control group received healthy recommendations to eat more fruit and vegetables and avoid refined grains and red meat. Adherence to the MeDi was monitored by a 24 h recall every 2 weeks, weekly follow-up via phone calls and in-office counseling. Following the intervention, the MeDi group significantly improved in terms of the MoCA total score (*p* = 0.001) and its individual components of executive function (*p* = 0.001), language (*p* = 0.01), attention, concentration and working memory (*p* = 0.04) compared to the control group. In contrast, the control group was significantly worse in terms of the MoCA total score (*p* = 0.04). No change was found for the components of visuospatial abilities, short-term memory recall and orientation to time and place.

Another RCT by Tsolaki and colleagues, named the MICOIL study, examined the comparative effects of MeDi alone or supplemented with 50 mL of high phenolic EVOO (HP-EVOO) per day or 50 mL of moderate phenolic EVOO (MP-EVOO) per day and analyzed 50 individuals with MCI via the Peterson criteria with a mean age of 69.8 ± 6.9 years and a baseline mean MMSE score of 27.5 ± 1.8 [65]. The participants were randomly allocated to the MeDi-HP-EVOO group (18 participants), to the MeDi-MP-EVOO group (16 participants), or were given instructions for the MeDi (16 participants). Adherence to each diet was secured by free administration of the EVOO with a 50 mL measuring device and regular contact with a doctor and dietician during the study team. After 12 months of the intervention, the MeDi-HP-EVOO group significantly improved in terms of ADAS-Cog (*p* = 0.003), LFT (*p* = 0.003) and F-DS (*p* = 0.006) test. In comparison, the MeDi-MP-EVOO group significantly improved on the MMSE (*p* = 0.01) and ADAS-Cog (*p* = 0.001), while the MeDi group significantly improved on the LFT (*p* = 0.02). The MeDi-HP-EVOO group performed significantly better on ADAS-Cog in comparison to the MeDi-MP-EVOO group (*p* = 0.03) and the MeDi group (*p* = 0.02), as well as on the LFT in comparison to the MeDi-MP-EVOO group (*p* = 0.002) and on the F-DS test in comparison to the MeDi group (*p* = 0.02). Lastly, the MeDi-MP-EVOO group performed significantly better on ADAS-Cog in comparison to the MeDi group (*p* = 0.001). No significant change was found in participants’ immediate and delayed story recall, or on the ROCFT, TMT-A&B, B-DS test, CFT and CDT. A further analysis of the participants with at least one APOE ε4 allele revealed a significant improvement of the MeDi-HP-EVOO group on MMSE compared to that of the MeDi-MP-EVOO group (*p* = 0.04), while the previously significant changes in the F-DS test were lost. All others reported significant changes remained. Overall, the type of dietary intervention predicted the difference in the ADAS-Cog score between the baseline and post-intervention at 12 months (*p* = 0.03).

Wardle and colleagues designed an RCT to test whether cholesterol-lowering diets adversely affect cognitive function [66]. The participants analyzed were 155 cognitively normal adults with a mean age of 53 ± 10.2 years, who had raised levels of serum cholesterol (>198 mg/dL), but no other serious health problems. Participants were randomly assigned to a LF diet (55 participants), a MeDi (53 participants), or a waiting-list control group (50 participants) for 12 weeks. Adherence was monitored by a seven-day dietary diary at the baseline and at the endpoint for the follow-up. At the follow-up, the MeDi group performed significantly better on the sustained attention task (SAT) and the Bakan task in comparison to the other groups (*p* < 0.001). No significant differences were detected between the groups in verbal immediate free recall, tapping speed and choice reaction time.

### 3.1. Ketogenic Diet (KD)

**Figure 2:**
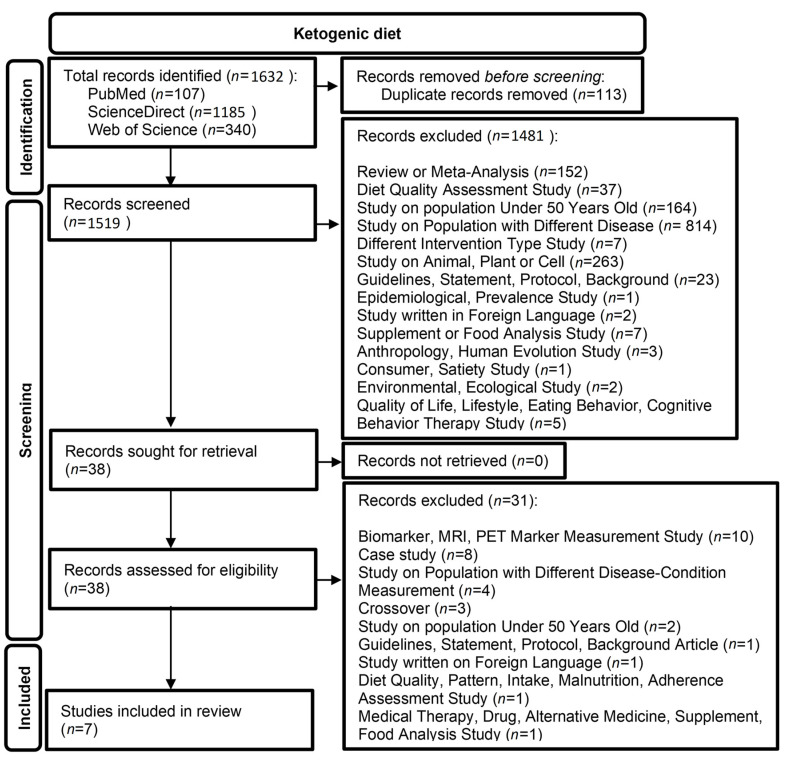
Flow chart of the literature search for articles on the ketogenic diet (KD).

In total, 1632 publications were identified that referred to the KD. The flow chart of the literature search is presented in Figure 2. After removing 113 duplicates, 1519 studies were screened and assessed for eligibility. Next, 1481 of the 1519 articles were excluded after reading the titles and abstracts, and the remaining 38 full-text articles were estimated for their eligibility. Eventually, seven studies were included, all published between 2000 and 2021. Table 4 and Table 5 show the quality assessment of included studies, as shown in Table 6.

The periods of the KD interventions varied between 21 days and 6 months. The total number of participants was 313: in five studies the participants had been diagnosed with MCI; and in two studies they had been diagnosed with AD. Out of the seven studies, six reported beneficial effects of the KD intervention on cognitive function, reaching statistical significance for at least one cognitive test in a subgroup of participants. Low adherence to the KD and a small sample size were reported in the only study in which the effects on cognition did not reach statistical significance.

Brandt and colleagues conducted a 12-week RCT that included participants with MCI or early AD; specifically, there were nine participants in the intervention group and five in the control group, with a mean age of 71.9 ± 6.3 years and a mean MoCA score of 20.4 ± 3.8 [67]. The diagnosis for MCI or early AD followed the National Institute on Aging and Alzheimer’s Association’s (NIA-AA) diagnostic guidelines. For the intervention group, a modified Atkins Diet (MAD) was applied, in which the net intake of carbohydrates was limited to 20 g or less, the fat intake was high, the protein intake was moderate and hydration was adequate. The control group followed the NIA diet recommendations, which emphasize the consumption of vegetables, fruits, whole foods and low-fat products. Both groups took a multivitamin, as well as calcium and vitamin D supplements. Adherence was measured by daily urine ketone testing with 67% of the intervention group achieving urine ketone levels ≥ 0.86 mmol/L at any point in the study and by food records with 44% of intervention group participants achieving a daily carbohydrate intake of ≤35 g. None of the control group participants achieved adequate adherence to the control diet with a Healthy Eating Index (HEI) of ≥85. No significant changes in cognition were observed in either group after the intervention, using the Hopkins Verbal Learning Test-Revised (HVLT-R), Brief Visuospatial Memory Test-Revised (BVMT-R) and the expanded MMSE version 2. When analysed in terms of ketone level, the MAD diet participants who achieved urine ketone levels ≥0.86 mmol/L had significantly improved their memory outcomes at week 6 of the intervention in contrast to the participants who did not (*p* = 0.03).

Fortier and colleagues performed a six-month RCT as part of the Brain ENErgy Fitness, Imaging and Cognition (BENEFIC) trial, with 19 people in the intervention group and 20 in the control group, with a mean age of 74.6 ± 6.5 years and with mean scores on MoCA of 22.9 ± 3 and MMSE of 27.4 ± 2.2 [68]. The participants were diagnosed with MCI via the Peterson criteria. Patients with DM were excluded. The participants received either a ketogenic MCT drink supplement, containing 30 g of MCT, 60% caprylic acid and 40% capric acid in 250 mL bottles or an isocaloric-matched placebo drink. No further advice to change participants’ habitual diets was given. The active or placebo drink to be consumed was split evenly between two meals. Compliance was on average 90% ± 8% via the monthly empty bottle count, while the blood acetoacetate levels increased on average from 0.12 mmol/L to 0.27 mmol/L (*p* < 0.001) and the βHB levels from 0.2 mmol/L to 0.54 mmol/L (*p* < 0.001) at the end of the study as compared to those at the baseline in the intervention group. The only statistically significant intergroup effects were found on language in terms of the BNT for total correct responses (*p* = 0.003). Significant beneficial intragroup effects in the intervention group were found on their episodic memory in terms of the Free and Cued Word Learning and Recall Test (F&C-WL&RT) (*p* = 0.013) and the BVMT-R (*p* = 0.027), and on their attention via the Stroop test in terms of the number of self-corrected (*p* = 0.046) and noncorrected errors (*p* = 0.036) and the TMT in terms of the number sequencing (*p* = 0.043). In contrast, the control group worsened on language as shown by the delayed total recall test (*p* = 0.026), on executive function as shown by the CFT (*p* = 0.47) and on processing speed as shown by the TMT (*p* = 0.022), while the intervention group remained unchanged. The MMSE and MoCA scores did not change (*p* > 0.1).

Additionally, Fortier and colleagues conducted another six-month RCT with 83 MCI patients diagnosed based on Peterson criteria [69]. The intervention group included 39 patients and the control group included 44 patients, with a mean age of 72.2 ± 7.1 years and with mean scores on MoCA of 24 ± 2.5 and MMSE of 27.5 ± 2.2. The same MCT drink intervention and trial design was used as described previously. Compliance was on average 89% ± 9% as determined via the monthly empty bottle count, while the blood acetoacetate levels increased on average from 0.09 mmol/L to 0.2 mmol/L (*p* < 0.001), the βHB levels from 0.15 mmol/L to 0.4 mmol/L (*p* < 0.001) and the total ketones from 0.24 mmol/L to 0.6 mmol/L (*p* < 0.001) at the end of the study as compared to the baseline in the intervention group. There was a statistically significant adjusted difference in episodic memory as shown by the first free recall trial of the F&C-WL&RT (*p* = 0.047), in executive function as shown by VF (*p* = 0.024) and the fewer errors in the TMT (*p* = 0.017) and Stroop test (*p* = 0.042), as well as in language as shown by the BNT (*p* = 0.033). These changes correlated significantly with the total plasma ketones (*p* < 0.05). In contrast, the total free recall, BVMT-R, MoCA and MMSE did not achieve statistical significance.

Henderson and colleagues performed a 90-day, randomized, double-blind, placebo-controlled, parallel-group study of 96 intervention-compliant patients diagnosed with mild to moderate AD, according to the National Institute of Neurological and Communicative Disease and Stroke, the Alzheimer’s Disease and Related Disorder Association (NINCDS-ADRDA), and DSM-IV criteria with a mean MMSE score of 19.6 ± 4.4 [70]. The intervention group consisted of 46 patients and the control group of 50 patients with a mean age of all baseline participants of 76.9 ± 8.3 years. Patients with DM were excluded. The participants in both groups followed their usual diet, but those in the intervention group also took AC-1202, which is an MCT composed of glycerin from vegetable oil and caprylic acids from coconut or palm kernel oil. The investigated product was given as a powder packaged in 30 g sachets containing either 10 g of MCT, 19.25 g of Acacia gum and 7.75 g of syloid or an isocaloric-matched placebo. The powder was mixed with water, milk or juice and consumed during breakfast. The dosage started with 10 g of MCT daily for the first week and increased to 20 g of MCT daily until the end of the study. No other advice to change participants’ usual diets was given. MCT intake induced transient ketosis, reaching on average blood βHB levels of 0.3–0.4 mmol/L 2 h after ingestion, even after carbohydrate-rich meals. The measures of cognition used were ADAS-Cog, the Cooperative Study-Clinical Global Impression of Change (ADCS-CGIC) and MMSE. On ADAS-Cog, a significant difference between the groups was observed on day 45 (*p* = 0.02) but no change was observed on day 90 (*p* = 0.06); moreover, no differences were observed on the ADCS-CGIC and MMSE scales. A further analysis on patients who lacked the APOE ε4 alleles revealed a statistically significant improvement on ADAS-Cog on day 45 (*p* = 0.001) and day 90 (*p* = 0.006), as well as on ADCS-CGIC on day 45 (*p* = 0.05) but no change on day 90 (*p* = 0.46) when compared to the control or the group with the APOE ε4 allele present. The presence of APOE ε4 may play a role in the efficacy of MCT intake on cognition.

Krikorian and colleagues conducted an RCT of 6 weeks, including 23 participants with MCI, a mean age of 69.4 ± 6.1 years and a mean CDR sum boxes score of 0.7 ± 0.4 [71]. Participants were randomized with 12 participants who followed a low-carbohydrate diet (the intervention group) and restricted their daily carbohydrate intake to ≤ 20 g and 11 participants who followed a high-carbohydrate diet (the control group) with ≥50% of their daily calories consumed from carbohydrates. Intervention adherence was monitored by weekly contact with participants, daily food records with a mean daily consumption of 34 ± 18 g carbohydrates, and urine ketone levels after overnight fasting with levels of 0.93 ± 0.54 mmol/L in the intervention group. The memory performance of the intervention group significantly improved as shown by the VPAT (*p* = 0.01) which was positively correlated to the urine ketone levels (*p* = 0.04). No significant changes were found in the TMT-B.

Krikorian and colleagues also conducted an 8-week RCT that included 14 PD patients, according to the UK Brain Bank criteria, with cognitive signs and symptoms corresponding to MCI according to the Movement Disorder Society Task Force guidelines for Level I PD-MCI [72]. The intervention and control group consisted of seven patients each with a mean age of 65.7 ± 6 years and a mean MoCA score of 24.8 ± 2. The only nutrient that was different between the two groups was the carbohydrates; the investigation group was limited to 20 g of carbohydrates per day, exclusively from vegetables. The control group was given a high-carbohydrate diet, designed to maintain their overall carbohydrate intake at the level typical of a Western diet, and well above the level required to induce ketone production. Blood βHB levels were measured after overnight fasting during weeks 2, 4 and 6 to assess participants’ adherence, which was 0.08 ± 0.01 mmol/L in the control group in contrast to 0.31 ± 0.19 mmol/L in the KD group (*p* = 0.01). After the eight-week intervention, the KD group showed significant improvement on both the Controlled Oral Word Association test (COWA) (*p* = 0.02) and on the VPAT (*p* < 0.04), while a beneficial trend was observed in the California Verbal Learning Test (CVLT) (*p* = 0.06).

De la Rubia Ortí and colleagues conducted an RCT on patients diagnosed with AD, institutionalized in the Alzheimer’s Family Association of Valencia (AFAV) [73]. Of the 44 patients, 22 followed a MeDi, enriched with 40 mL of coconut oil daily with 15% proteins, 55% carbohydrates, 30% lipids (coconut oil) and 20–30 g per day of soluble and insoluble fibre. The coconut oil was administered by blinded health workers from the institution. No further monitoring of ketone levels was conducted. The other 22 patients followed a MeDi with 15% proteins, 55% carbohydrates and 30% lipids. Cognition was measured by a seven-minute screening consisting of four tests: Benton’s Temporal Orientation test, the Clock Drawing Test, categorical Verbal Fluency test, and Free and Cued Selective Reminding Test. Significant benefits were found in the intervention group for temporal orientation in mild to moderate AD (*p* < 0.05), for semantic memory in females with mild to moderate AD and in males with severe AD (*p* < 0.05), and for episodic memory in participants with mild to moderate AD (*p* < 0.05).

### 3.2. MIND Diet

**Figure 3:**
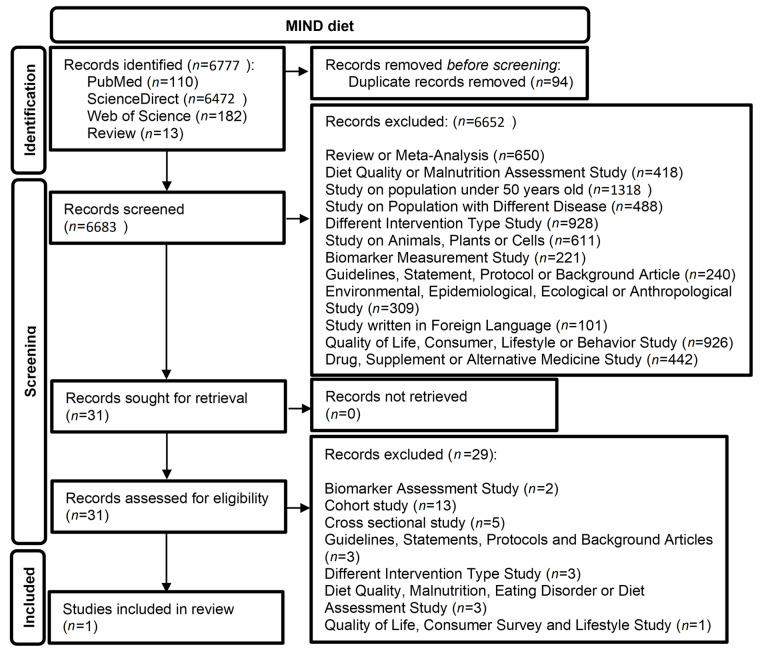
Flow chart of the literature search for articles on the MIND diet.

In total, 6764 publications were identified (PubMed: 110, ScienceDirect: 6472, Web of Science: 182). Additionally, 13 studies were identified by another systematic review [48]. After removing 94 duplicates, 6683 articles were screened and assessed for eligibility, of which 6652 were excluded by screening the abstracts. The remaining 31 articles were retrieved and their full texts were screened, after which 30 articles were excluded. Only one study was included in the final review. Table 7 and Table 8 show the quality assessment of the included study, as presented in Table 9. The flow chart of the literature search is presented in Figure 3.

Arjmand and colleagues performed an RCT study in Iran, the only intervention study on MIND included here that investigated the effects of a three-month MIND diet intervention on the cognitive effects of healthy obese women with a mean body mass index (BMI) of 32 ± 1 [74]. The study included 37 subjects, of which 22 were in the MIND diet intervention group and 15 in the control group with a mean age of 48.9 ± 1.3 years and a baseline MMSE of 26.4 ± 0.5. The dietary intake of participants was measured using a 168-item semi-quantified FFQ and participants’ adherence was monitored by the MIND diet score and three-day food recall every week. As calculated by the provided mean food consumption by item, at the baseline both groups had a mean MIND diet score of 7, and by the endpoint at 3 months, the intervention group had a MIND diet score of 10.5, an increase of 2.5, whereas the MIND diet score of the control group remained at 7. Wine intake, the 15th food item of the MIND diet score, was not taken into account since alcohol consumption is prohibited by law in the country conducting the trial. The MIND diet intervention group significantly improved participants’ working memory as shown by the LNS (*p* ≤ 0.001), their verbal recognition memory as shown by the RAVLT (*p* ≤ 0.001), their verbal short memory as shown by the F-DS (*p* ≤ 0.001) and B-DS (*p* = 0.041) tests, and their attention and visual scanning as shown by the SS&C (*p* ≤ 0.001) compared with the control group. Mixed results were observed in participants’ executive function with a statistically significant improvement in TMT-A (*p* = 0.002) but not in TMT-B (*p* = 0.161), whereas no benefit was observed in their ability to inhibit cognitive interference as shown by the Stroop task (*p* = 0.128).

## 4. Discussion

The aim of this systematic review was to collect and compare the current data on the effect of three dietary patterns, the MeDi, KD and MIND diets, on the prevention and progression of dementia. All three dietary interventions were documented to be beneficial in preventing cognitive impairment in specific and diverse domains.

The MeDi has been studied extensively and has been shown to provide health benefits as part of the general Mediterranean lifestyle, which is a social lifestyle, with the family as its epicentre; their way of life is more relaxed and stress-free, with regular midday sleep or rest sessions, outdoor activities close to nature, and frequent exposure to sunlight [75]. Adopting the Mediterranean lifestyle as a general approach can have synergistic effects on dementia prevention, leading to better compliance with the MeDi and, therefore, to higher MeDi scores [76]. With the exception of two studies, all the reviewed RCTs on the MeDi showed a significant improvement in at least one domain of cognitive function [48,51,52,53,54,55,56,57,58,59,60,61,73]. Positive results were observed in healthy individuals and in those diagnosed with various forms of cognitive impairment. Both the MeDi and MIND have been associated with fewer depressive symptoms and the MeDi has been shown to offer protection against the development of depressive symptoms in participants of an older age [41,77].

Studies that examine the associations of the MeDi and the accumulation of cerebral amyloid-β (Aβ) using positron emission tomography (PET) imaging in older adults have shown that higher MeDi scores, meaning high vegetable consumption and high dietary vitamin A and β-carotene intake with moderate alcohol consumption, correlate with low Aβ plaque formation, less cerebral Aβ accumulation and a better neuroimaging biomarker profile [78,79].

Adherence to the MeDi has been associated with specific alterations in gut microbiota. The NU-AGE one-year dietary intervention showed that MeDi adherence correlated with changes in gut flora composition, lower frailty, improved cognitive function and a reduction in inflammatory markers, such as C-reactive protein (CRP) and interleukin-17 (IL-17) [80]. Interestingly, improved global cognitive function has been reported also in adults with type 2 DM with high MeDi adherence [81]. The combination of increased physical activity and higher MeDi adherence was shown to be associated with decreased brain Aβ load and increased glucose metabolism among cognitively normal individuals, suggesting that both could be used as possible counter measures against the decline in cognitive function [82].

Higher adherence to the MeDi can improve cognitive impairment and is associated with better cognitive performance especially in older individuals [83]. In fact, MeDi adherence is one of the approaches recommended for nutrition therapy in CVD. Therefore, the MeDi can be used for patients with a high risk of CVD with cognitive impairment to provide a non-pharmacological path towards improving circulatory and cognitive function. Overall, the MeDi is a nutrition pattern that when followed closely, offering improvements in general health status and cognitive function. According to the analysis of the studies included in this review, patients with the highest MeDi scores show the best results for preventing cognitive impairment, similarly to the DASH and MIND diets.

The KD is used primarily for patients with drug-resistant epilepsy, but it also has beneficial effects in preventing cognitive impairment [35]. The KD focuses on high fat and low carbohydrate intake in order to achieve ketosis, which can be measured by assessing ketone bodies in urine and blood samples. Because of the strict restriction of carbohydrates, the KD is not an easy protocol for patients with AD to follow, and in recent years, studies have examined the production of ketone bodies by supplementation with MCT [84]. The KD can be dangerous for patients with abnormal lipid metabolism, atherosclerosis, those who have a history or are at high risk of CVD, and those with osteoporosis, kidney stones and renal failure [44]. The effects of KD have been studied in overweight patients, and patients with DM and IR who could benefit in multiple ways besides the prevention of cognitive decline [39]. The duration of the KD intervention in the reviewed studies ranged from 21 days to 34 weeks. A longer maintenance of the KD could be considered with adequate medical monitoring.

A more subtle approach is a ketogenic MeDi that combines the two diets. In this protocol, the basis of the MeDi, namely, high vegetable and fruit consumption, remains the same, but the carbohydrates are restricted, and fat and protein consumption are increased to a degree that mild ketosis is achieved. Such diets are the modified Mediterranean–ketogenic diet (MMKD) and the Spanish ketogenic Mediterranean diet (SKMD) [85,86]. There is not yet sufficient research to have a clear view on the effects of a ketogenic MeDi in preventing cognitive decline, but the results to date are promising. The MMKD has shown beneficial effects on AD markers in the gut microbiome and the cerebrospinal fluid (CSF) in patients with MCI, while the SKMD has shown beneficial effects in a range of prevention targets, including obesity, CVD, metabolic syndrome, fatty liver disease and depression. Monitoring such diets requires the combination of MeDi adherence scoring and ketone body measurements in the urine or blood. The negative effects of the KD are tempered by the basis of the MeDi. It is still not known if a ketogenic MeDi is safe to use on the subgroup of patients that can be harmed by the KD. Patients who comply with the MeDi could benefit by diversifying their diet to a ketogenic MeDi, and in this context, patients struggling with weight loss are ideal candidates. The ketogenic MeDi, when adopted as a monophasic diet, can be maintained as long as the KD or longer, if a biphasic diet with the MeDi and ketogenic MeDi are alternatively used.

The DASH diet was developed to prevent hypertension and CVD, which are also targets of dementia prevention. Studies have shown that cognitive impairment can be significantly prevented only in the groups with the highest DASH diet score. A more promising diet for dementia prevention, as shown by the Rush Memory and Aging Project (MAP) studies, is its Mediterranean equivalent, the MIND diet. Higher compliance with the MIND diet, or a higher MIND diet score, is associated with a lower incidence of dementia in a linear fashion [87]. This effect could be observed only in the groups with very high compliance with the other dietary interventions. The MIND diet has also shown beneficial effects on mood, depressive symptoms and in performing daily activities, leading to a self-sufficient and independent lifestyle in the elderly [77,88]. Adherence to the MIND diet increases brain resilience to cognitive decline despite the underlying brain pathology [89]. These effects still need to be confirmed by successfully designed RCTs. In this review we found only one RCT that measured the cognitive effects of a MIND diet intervention. Early findings from observation and ongoing studies on MIND in cognition have shown promising results [45,46,90,91]. Nevertheless, it is recommended that the MIND diet should be adopted when possible, taking into consideration the high price of some of the food items included, such as olive oil, nuts, berries, and fish, especially off-season and for low- or middle-income patients in countries without domestic production of these food items. This counterintuitive effect of income on adherence to the MIND diet for cognition is observed in the Estudo Longitudinal de Saúde do Adulto (ELSA)-Brasil study [92]. The same restrictions apply for the MeDi, as both diets have many similarities.

Investigation into the effect of other dietary interventions on cognitive performance and mood should be encouraged. Such diets include the Okinawa diet, NU-AGE diet and the Nordic diet [58,93,94]. In addition, innovative diets, such as a ketogenic MIND diet or an updated modified MIND diet with new food items, such as saffron and curcumin, which have been sufficiently studied for their neuroprotective properties, should be considered [95,96]. One such diet is the KetoFLEX 12/3 which incorporates a light ketogenic effect and the principles of the MIND diet [97]. Within this systematic review, no publications of such innovative diets could be found. Multidomain interventions, such as the Finnish Geriatric Intervention Study to Prevent Cognitive Impairment and Disability (FINGER), have shown the synergetic effects of diet in dementia prevention [98]. Another ambitious multidomain intervention is the South Korean study to prevent cognitive impairment and protect brain health through lifestyle intervention in at-risk elderly people (SUPERBRAIN program), which reports that the incorporation of the MIND diet as part of the intervention has a beneficial effect on cognitive health [99,100]. Furthermore, the APOE gene status should be considered in dietary interventions on cognitive performance since APOE ε4 gene carriers show a greater cognitive benefit from a MeDi intervention but no cognitive benefit from KD interventions [60,65,70]. The interaction of dietary interventions, APOE gene status and cognitive performance is inconclusive, and therefore further investigation is needed.

### Strengths and Limitations

The present systematic review does not come without limitations. The number of databases searched was restricted to three: PubMed, ScienceDirect and Web of Science. The publications selected were restricted to the English language. Only one RCT could be found on the MIND diet as an intervention study. The data in the selected publications were too diverse, particularly those regarding the cognitive status of the intervention groups and the multiplicity of the neurocognitive tests used, for a meta-analysis to be conducted.

As for its strengths, however, this systematic review is the first to undertake a synthesized review of multiple dietary interventions for dementia prevention. No time restrictions were set in our database searches. The authors are health professionals covering different disciplines, including dieticians, medical doctors and pharmacists.

## 5. Conclusions

As this systematic review shows, dietary interventions can be beneficial in maintaining cognitive health, they can be integrated in the everyday practice of prevention clinics and be considered in national prevention programs. An individualised dietary intervention is proposed for each patient, according to an individual’s overall health status and personal characteristics, and the recommendation of a specific diet without considering the patient’s specific needs should be avoided.

## Figures and Tables

**Table 1:** Quality assessment of MeDi RCTs with RoB23.1 Mediterranean Diet (MeDi).

	Bias Arising from the Randomization Process	Bias Due to Deviations from Intended Intervention	Bias Due to Missing Outcome Data	Bias in Measurement of the Outcome	Bias in Selection of the Reported Results	Overall
Hoscheidt, 2021 [56]	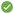	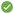	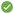	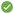	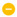	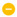
Knight, 2016 [57]	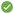	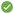	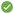	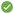	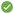	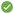
Marseglia, 2018 [58]	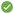	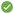	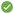	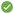	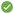	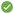
Martinez-Lapiscina, 2013a [59]	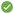	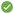	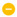	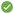	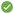	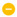
Martínez-Lapiscina, 2014 [60]	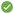	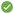	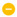	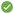	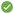	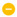
Martinez-Lapiscina, 2013b [61]	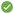	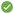	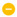	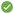	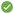	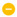
Valls-Pedret, 2015 [62]	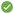	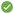	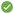	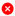	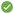	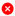
Mazza, 2018 [63]	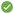	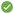	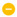	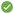	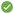	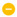
Paknahad, 2020 [64]	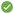	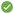	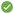	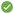	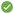	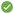
Tsolaki, 2020 [65]	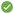	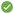	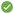	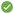	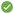	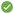
Wardle, 2000 [66]	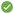	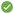	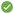	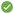	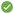	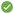

Judgment: 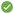 low bias; 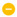 some concerns; 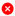 high bias.

**Table 2:** Quality assessment of MeDi RCTs with Jadad score.

	If Randomization Is Mentioned	If Randomization Method Is Appropriate	If Blinding Is Mentioned	If Blinding Method Is Appropriate	If (n Account) the Fate of All Patients Is Known	Overall Score	Overall Score Evaluation
Hoscheidt, 2021 [56]	1	0	1	1	0	3	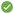
Knight, 2016 [57]	1	1	1	1	0	4	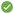
Marseglia, 2018 [58]	1	1	1	1	0	4	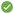
Martinez-Lapiscina, 2013a [59]	1	1	1	1	0	4	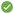
Martínez-Lapiscina, 2014 [60]	1	1	1	1	0	4	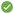
Martinez-Lapiscina, 2013b [61]	1	1	1	1	0	4	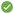
Valls-Pedret, 2015 [62]	1	1	0	0	0	2	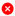
Mazza, 2018 [63]	1	0	1	1	0	3	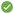
Paknahad, 2020 [64]	1	1	1	1	0	4	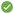
Tsolaki, 2020 [65]	1	1	1	1	0	4	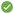
Wardle, 2000 [66]	1	0	1	1	0	3	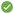

Overall Score Evaluation: 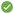 high quality; 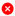 low quality.

**Table 3:** Characteristics of the reviewed studies on the effects of the Mediterranean Diet (MeDi) and its cognitive effects.

Study (REF) Year, Country	Type of Study	Intervention Group 1 Included	Intervention Group 2 Included	Control Group Included	Cognitive Status at Baseline	Overall Health Status	Intervention	Duration of Intervention	Measures of Cognitive Outcomes	Outcomes—Significant Benefit of Intervention
Hoscheidt [56] 2021 USA	RCT	*n*= 44 Age 55.7 ± 5.5 y	N/A	*n* = 43 Age 56.9 ± 4.7 y	NC 3MS 97 ± 2 & MCI 3MS 95 ± 4	Healthy	IG1: MeDi (<7% SF, GI < 55, Na^+^ 1.3 g/day) CG: Western diet (25% SF, GI > 70, Na^+^ 3.2 g/day)	4 weeks	3MS, SRT, BSRT, DCT	*p* > 0.1
Knight [57] 2016 Australia	RCT	*n* = 70 Age 72.1 ± 4.9 y	N/A	*n* = 67 Age 72 ± 5 y	NC	Healthy	IG1: MeDi CG: no guidance	6 months	Stroop, LFT, TOL, RAVLT, F&B-DS, LNS, SS&C, BVRT	BVRT (*p* = 0.01) RAVLT (*p* = 0.03) RAVLT/F&B-DS/LNS (*p* = 0.05)
Marseglia [58] 2018 France, Italy, Netherlands, Poland, and UK	RCT	*n* = 573 Age 70.7 ± 0.2 y	N/A	*n* = 571 Age 71.1 ± 0.2	NC MMSE 28 ± 2	Healthy	IG1: NU-AGE diet CG: national guidelines	12 months	CERAD-NB, MMSE, CFT, BSR, PCT, DCT, TMT-A&B, BNT, WLM, CPT	MMSE/CERAD-NB (*p* = 0.05) WLM/BSRT (*p* = 0.03)
Martinez-Lapiscina [59] 2013a Spain	RCT	*n* = 224 Age 67.4 ± 5.7 y	*n* = 166 Age 67.3 ± 5.8 y	*n* = 132 Age 67.6 ± 5.5 y	NC	High Risk of CVD	IG1: MeDi-EVOO (1 L/week) IG2: MeDi-MN (30 g/day) CG: LF	6.5 years	MMSE, CDT	MMSE (*p* = 0.015) CDT (*p* = 0.048)
Martínez-Lapiscina [60] 2014 Spain	RCT	*n* = 381 Age 67 ± 6 y	N/A	*n* = 129 Age 67 ± 6 y	NC	High Risk of CVD	IG: MeDi CG: LF	6.5 years	MMSE, CDT	CLU MMSE (*p* = 0.04) CLU CDT (*p* = 0.001) CR1 MMSE (*p* = 0.001) CR1 CDT (*p* = 0.006) PICALM MMSE (*p* = 0.02) PICALM CDT (*p* = 0.005) APOE MMSE (*p* < 0.001) APOE CDT (*p* = 0.007)
Martinez-Lapiscina [61] 2013b Spain	RCT	*n* = 91 Age 67.2 ± 5.6 y	*n* = 88 Age 67.3 ± 6 y	*n* = 89 Age 67.5 ± 5.7 y	NC	High Risk of CVD	IG1: MeDi-EVOO (1 L/week) IG2: MeDi-MN (30 g/day) CG: LF	6.5 years	MMSE, CDT, VPA, RAVLT, ROCFT, BNT, AFT, FAST, F&B-DS, TMT-A&B, CDR	MMSE, ROCF, FAST, F-DS (*p* < 0.05)
Valls-Pedret [62] 2015 Spain	RCT	*n* = 127 Age 67.9 ± 5.4 y	*n* = 112 Age 66.7 ± 5.3 y	*n* = 95 Age 65.5 ± 5.8 y	NC MMSE 28 ± 1	High Risk of CVD	IG1: MeDi-EVOO (1 L/week) IG2: MeDi-MN (30 g/day) CG: LF	4.1 years	MMSE, RAVLT, VPAT, AFT, F&B-DS, CTT-1&2	RAVLT/VPAT (*p* = 0.04) DS/CTT (*p* = 0.004) all tests composite (*p* = 0.01)
Mazza [63] 2018 Italy	RCT	*n* = 55 Age 70 ± 4 y	N/A	N = 55 Age 70 ± 4y	MCI MMSE 25 ± 1	52% HL 52% HT 48% DM	IG: MeDi-EVOO (20–30 g/day) CG: MeDi	12 months	MMSE, ADAS-Cog, VF	MMSE (*p* < 0.001) ADAS-Cog (*p* < 0.001)
Paknahad [64] 2020 Iran	RCT	*n* = 35 Age 59.3 ± 8.3 y	N/A	*n* = 35 Age 58.6 ± 9.3 y	PD-MCI MoCA 19 ± 6	PD	IG: MeDi CG: healthy recommendations	10 weeks	MoCA	total score (*p* = 0.001) executive function (*p* = 0.001) language (*p* = 0.02) attention, concentration, working memory (*p* = 0.04)
Tsolaki [65] 2020 Greece	RCT	*n* = 18 Age 68.5 ± 6.8 y	*n* = 16 Age 70.8 ± 8.1 y	*n* = 16 Age 70.1 ± 6 y	MCI MMSE 28 ± 2	Healthy	IG1: MeDi-HP-EVOO (50 mL/day) IG2: MeDi-MP-EVOO (50 mL/day) CG: MeDi	12 months	MMSE, SRT, ROCF, TMT-A&B, ADAS-Cog, F&B-DS, LFT, CFT, CDT	ADAS-Cog (*p* = 0.001) MMSE (*p* = 0.03) F-DS (*p* = 0.006) LFT (*p* = 0.003)
Wardle [66] 2000 United Kingdom	RCT	*n* = 52 Age 52 ± 11 y	*n* = 53 Age 54 ± 11 y	*n* = 50 Age 53 ± 8 y	NC	HL	IG1: LF IG2: MeDi CG: no guidance	12 weeks	VIFR, TFT, CRT, SAT	SAT (*p* < 0.001)

3MS, modified Mini Mental Status Examination; ADAS-Cog, Alzheimer’s Disease Assessment Scale—Cognitive; AFT, Animal Fluency Test; APOE, Apolipoprotein; BNT, Boston Naming Test; BSR, Babcock Story Recall; BSRT, Buschke Selective Reminding Test; BVRT, Benton Visual Retention Test; CDR, Clinical Dementia Rating; CDT, Clock Drawing Test; CERAD-NB, Consortium to Establish a Registry for Alzheimer’s Disease neuropsychological battery; CFT, Category Fluency Test; CG, control group; CLU, CLU gene; CPT, Constructional Praxis Test; CR1, CR1 gene; CRT, Choice Reaction Time; CTT-1&2, Color Trail Test—part 1 and 2; CVD, cardiovascular disease; DC, digit cancellation; DCT, Dot Counting Test; DM, diabetes mellitus; EVOO, extra virgin olive oil; F&B-DS, forward and backward digit span test; FAST, F-A-S test; GI, glycemic index; HL, hyperlipidemia; HP-EVOO, high phenolic extra virgin olive oil; HT, hypertension; IG, intervention group; LF, low fat; LFT, Letter Fluency Test; LNS, Letter–Number Sequencing test; MCI, Mild Cognitive Impairment; MeDi, Mediterranean diet; MMSE, Mini Mental Status Examination; MN, mixed nuts (15 g walnuts + 7.5 g almonds + 7.5 g hazelnuts); MoCA, Montreal Cognitive Assessment; MP-EVOO, moderate phenolic extra virgin olive oil; N/A, not applicable; NC, normal cognition; PCT, Pattern Comparison Test; PD, Parkinson’s Disease; PD-MCI, Mild Cognitive Impairment in Parkinson’s Disease; PICALM, PICALM gene; RAVLT, Rey Auditory Verbal Learning Test; RCT, Randomized Controlled Trial; ROCFT, Rey-Osterreich Complex Figure; SAT, sustained attention test; SF, saturated fat; SRT, Story Recall Test; SS&C, Symbol Search and Coding test; Stroop, Stroop test; TFT, two-finger tapping speed; TMT-A&B, Trail Making Test-part A and B; TOL, Tower of London planning test; VF, Verbal Fluency; VIFR, Verbal Immediate Free Recall; VPA, Verbal Paired Association test; WLM, World List Memory; y, years.

**Table 4:** Quality assessment of Keto diet RCTs with RoB2.

	Bias Arising from the Randomization Process	Bias Due to Deviations from Intended Intervention	Bias Due to Missing Outcome Data	Bias in Measurement of the Outcome	Bias in Selection of the Reported Results	Overall
Brandt, 2019 [67]	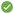	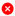	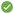	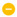	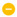	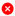
Fortier, 2019 [68]	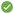	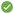	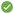	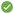	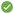	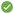
Fortier, 2021 [69]	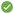	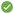	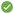	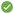	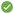	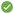
Henderson, 2009 [70]	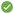	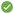	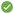	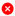	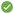	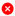
Krikorian, 2012 [71]	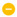	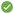	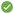	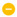	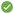	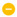
Krikorian, 2019 [72]	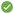	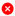	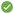	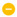	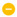	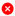
de la Rubia Ortí, 2018 [73]	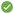	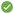	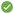	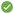	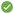	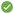

Judgment: 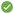 low bias; 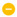 some concerns; 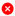 high bias.

**Table 5:** Quality assessment of Keto diet RCTs with Jadad score.

	If Randomization Is Mentioned	If Randomization Method Is Appropriate	If Blinding Is Mentioned	If Blinding Method Is Appropriate	If (n Account) the Fate of All Patients Is Known	Overall Score	Overall Score Evaluation
Brandt, 2019 [67]	1	1	1	1	0	4	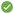
Fortier, 2019 [68]	1	1	1	1	0	4	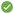
Fortier, 2021 [69]	1	1	1	1	0	4	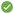
Henderson, 2009 [70]	1	1	1	1	0	4	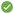
Krikorian, 2012 [71]	0	0	0	0	1	1	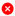
Krikorian, 2019 [72]	1	1	0	0	0	2	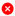
de la Rubia Ortí, 2018 [73]	1	1	1	1	0	4	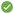

Overall Score Evaluation: 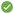 high quality; 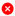 low quality.

**Table 6:** Characteristics of the reviewed studies on the ketogenic diet (KD) and its cognitive effects.

Study (REF) Year Country	Type of Study	Intervention Group Included	Control Group Included	Cognitive Status at Baseline	Overall Health Status	Intervention (Details of Diet)	Duration of Intervention	Measures of Cognitive Outcomes	Outcomes—Significant Benefit of Intervention
Brandt [67] 2019 USA	RCT	*n* = 9 Age 73.5 ± 6.4 y	*n* = 5 Age 69.1 ± 5 y	MCI or early AD MoCA 20.4 ± 3.8	NR	IG: MAD CG: NIA diet	12 weeks	CDR, MoCA, MMSE-2:EV, HVLT-R, BVMT-R	*p* > 0.05
Fortier [68] 2019 Canada	RCT	*n* = 19 Age 73.8 ± 6.3 y	*n* = 20 Age 75.4 ± 6.6 y	MCI MoCA 22.9 ± 3 MMSE 27.4 ± 2.2	NR	IG: 15 g MCT drink (60% caprylic acid, 40% capric acid) twice daily CG: Placebo sunflower oil drink	6 months	MMSE, MoCA, F&C-WL&RT, BVMT-R, TMT, Stroop, VF, DSST, BNT	BNT *p* = 0.003 F&C-WL&RT *p* = 0.013 BVMT-R *p* = 0.027 Stroop *p* = 0.046 TMT *p* = 0.043
Fortier [69] 2021 Canada	RCT	*n* = 39 Age 71.4 ± 7.2 y	*n* = 44 Age 72.9 ± 6.9 y	MCI MoCA 24 ± 2.5 MMSE 27.5 ± 2.2	NR	IG: 15 g MCT drink (60% caprylic acid, 40% capric acid) twice daily CG: Placebo sunflower oil drink	6 months	MMSE, MoCA, F&C-WL&RT, BVMT-R, TMT, Stroop, VF, DSST, BNT	F&C-WL&RT *p* = 0.047 VF *p* = 0.024 TMT *p* = 0.017 Stroop *p* = 0.042 BNT *p* = 0.033
Henderson [70] 2009 USA	RCT	*n* = 46 Age 76.9 ± 8.9 y	*n* = 50 Age 76.8 ± 7.4 y	Mild-moderate AD MMSE 19.6 ± 4.4	NR	IG: 20 g MCT drink (glycerin, caprylic acid) daily CG: Placebo safflower oil drink	90 days	ADAS-Cog, ADCS-CGIC, MMSE	ADAS-Cog *p* = 0.02
Krikorian [71] 2012 USA	RCT	*n* = 12 Age 68 ± 3 y	*n* = 11 Age 71 ± 8 y	MCI CDR 0.7 ± 0.4	NR	IG: ≤20 g carbohydrates per day CG: 50% of daily calories from carbohydrates	6 weeks	CDR, TMT-B, VPAL	VPAL *p* = 0.01
Krikorian [72] 2019 USA	RCT	*n* = 7 Age 66 ± 5.5 y	*n* = 7 Age 65.4 ± 6.5 y	PD-MCI MoCA 24.8 ± 2	NR	IG: ≤20 g carbohydrates per day CG: 50% of daily calories from carbohydrates	8 weeks	MoCA, COWA, CVLT, VPAL	COWA *p* = 0.02 VPAL *p* < 0.04 CVLT *p* = 0.06
de la Rubia Ortí [73] 2018 Spain	RCT	*n* = 22 Age 65-85 y	*n* = 22 Age 65-85 y	AD (institutionalized)	NR	IG: MeDi + 20 mL coconut oil twice daily CG: MeDi	21 days	MMSE, BTOT, CDT, VF, F&C-SRT	BTOT *p* < 0.05 VF *p* < 0.05 F&C-SRT *p* < 0.05

AD, Alzheimer’s Disease; ADAS-Cog, Alzheimer’s Disease Assessment Scale—Cognitive subscale; ADCS-CGIC, Alzheimer’s Disease Cooperative Study—Clinical Global Impression of Change; BNT, Boston Naming Test; BTOT, Benton’s Temporal Orientation Test; BVMT-R, Brief Visuospatial Memory Test—Revised; CDR, Clinical Dementia Rating; CDT, Clock Drawing Test; CG, control group; COWA, Controlled Oral Word Association Task; CVLT, California Verbal Learning Test; DSST, Digit Symbol Substitution Test; F&C-SRT, Free and Cued Selective Reminding Test; F&C-WL&RT, Free and Cued Word Learning and Recall Test; HVLT-R, Hopkins Verbal Learning Test—Revised; IG, investigation group; MAD, modified Atkins diet; MCI, mild cognitive impairment; MCT, medium chain triglycerides; MeDi, Mediterranean diet; MMSE-2:EV, Mini Mental Status Examination—2, Expanded Version; MoCA, Montreal Cognitive Assessment; NIA, National Institute on Aging; NR, not reported; PD, Parkinson’s Disease; RCT, randomized clinical trial; Stroop, Stroop Color and Word Interference; TMT-B, Trail Making Test, list B; VF, categorical Verbal Fluency test; VPAL, Verbal Paired Associate Learning Test; y, years.

**Table 7:** Quality assessment of MIND diet RCTs with RoB2.

	Bias Arising from the Randomization Process	Bias Due to Deviations from Intended Intervention	Bias Due to Missing Outcome Data	Bias in Measurement of the Outcome	Bias in Selection of the Reported Results	Overall
Arjmand, 2020 [74]	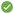	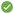	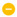	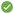	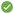	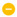

Judgment: 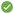 low bias; 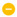 some concerns.

**Table 8:** Quality assessment of MIND diet RCTs with Jadad score.

	If Randomization Is Mentioned	If Randomization Method Is Appropriate	If Blinding Is Mentioned	If Blinding Method Is Appropriate	If (n Account) the Fate of All Patients Is Known	Overall Score	Overall Score Evaluation
Arjmand, 2020 [74]	1	1	1	1	0	4	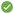

Overall Score Evaluation: 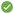 high quality.

**Table 9:** Characteristics of the reviewed studies on the MIND diet and its cognitive effects.

Study (REF) Year, Country	Type of Study	Intervention Group Included	Control Group Included	Cognitive Status at Baseline	Overall Health Status	Intervention	Duration of Intervention	Measures of Cognitive Outcomes	Outcomes—Significant Benefit of Intervention
Arjmand [74] 2020 Iran	RCT	*n* = 22 Age 49 ± 1.1 y	*n* = 15 Age 48.9 ± 1.6 y	CN MMSE 26.4 ± 0.5	Obesity BMI 32 ± 1	IG: calorie-restricted MIND diet CG: calorie-restricted diet	3 months	MMSE, F&BDST, LNST, SDMT, RAVLT, TMT-A&B, Stroop	LNST *p* ≤ 0.001 RAVLT *p* ≤ 0.001 F&BDST *p* ≤ 0.05 SDMT *p* ≤ 0.001 TMT-A *p* = 0.002

BMI, Body Mass Index; CG, control group; CN, cognitively normal; F&BDST, Forward and Backward Digit Span Task; IG, investigation group; LNST, Letter Number Sequencing Task; MIND, Mediterranean-DASH (Dietary Approaches to Stop Hypertension) Intervention for Neurodegenerative Delay; MMSE, Mini Mental Status Examination; RAVLT, Rey Auditory Verbal Learning Test; RCT, Randomized Controlled Trial; SDMT, Symbol Digit Modalities Test; Stroop, cognitive interference Stroop task; TMT-A&B, Trail Making Test-part A and B; y, years.

## Data Availability

Not applicable.

## Supplementary Materials

The following supporting information can be downloaded at: https://www.mdpi.com/article/10.3390/life13010173/s1, Table S1: PRISMA 2020 item checklist; Table S2: PRISMA 2020 for Abstracts checklist; Table S3: Search strings used in the systematic reviews. Reference [101] was cited in the supplementary materials.
